# Automated Classification of Second- and Third-Degree Burn Images Using Convolutional Neural Networks

**DOI:** 10.3390/ebj7020033

**Published:** 2026-06-10

**Authors:** Yamile Montecinos-Rodríguez, Francisco J. Torres-Santana, Noureddine Lakouari, Lorena Díaz-González

**Affiliations:** 1 Licenciatura en Inteligencia Artificial, Instituto de Investigación en Ciencias Básicas y Aplicadas (IICBA), Universidad Autónoma del Estado de Morelos, Cuernavaca 62209, Morelos, Mexico; yamile.montecinos@uaem.edu.mx (Y.M.-R.); francisco.torressa@uaem.edu.mx (F.J.T.-S.); 2 Instituto Nacional de Astrofísica, Óptica y Electrónica, Luis Enrique Erro 1, Tonantzintla 72840, Puebla, Mexico; n.lakouari@inaoep.mx; 3 Secretaría de Ciencia, Humanidades, Tecnología e Innovación (SECIHTI), Insurgentes Sur 1582, Ciudad de México 03940, Mexico; 4 Centro de Investigación en Ciencias, Universidad Autónoma del Estado de Morelos, Cuernavaca 62209, Morelos, Mexico

**Keywords:** burn severity classification, burn degree, convolutional neural networks, deep learning, medical image analysis, computer-assisted diagnosis, clinical decision support

## Abstract

**Highlights:**

**What are the main findings?**

- A compact convolutional neural network trained from scratch accurately classified second- and third-degree burn images from clinical photographs.
- The green color channel provided the most informative input and achieved higher performance than RGB-based representations.

**What are the implications of the main findings?**

- Accurate burn severity classification can be achieved using a lightweight deep learning model without relying on complex transfer learning architectures.
- This approach may support rapid burn assessment and contribute to the development of accessible clinical decision-support tools.

**Abstract:**

Background: Burn severity assessment is clinically relevant and often requires timely decision-making. Visual classification of second- and third-degree burns remains subjective and prone to observer variability. Methods: This study aimed to develop and evaluate a deep learning model for the automatic classification of second- and third-degree burn images. A dataset of clinical burn images from a private wound care clinic was used to train a convolutional neural network. Hyperparameter optimization and color channel sensitivity analysis were performed to identify the optimal model configuration. Model performance was evaluated using standard classification metrics on training, validation, and independent test datasets, and results were compared with transfer learning approaches. Results: The best performance was achieved using a compact model trained exclusively with the green color channel, obtaining an accuracy of 0.94, precision of 0.96, recall of 0.92, and an F1-score of 0.94 on the independent test set. This model outperformed more complex transfer learning approaches while reducing computational complexity. Conclusions: These findings indicate that using only the green color channel enables efficient and accurate burn classification. The proposed model was also integrated into a graphical user interface, supporting its potential application in clinical and educational environments.

## 1. Introduction

Burns are a major public health problem. Each year, about 180,000 deaths occur due to burn injuries, mostly in low- and middle-income countries [1]. Many patients require specialized care from the acute phase through long-term rehabilitation [2]. Burns are tissue injuries caused by exposure to heat, radiation, radioactivity, electricity, friction, or chemical exposure. These agents increase the temperature of the skin and underlying tissues, leading to cell death or tissue carbonization [3]. Burns can be classified based on wound depth, extent, and the percentage of total body surface area (TBSA) affected, and together, these factors determine burn degree [4].

- First-degree (FD): These are superficial, affecting only the epidermis. The skin appears dry, red, and painful, but heals without treatment and does not leave scars [5].
- Second-degree (SD): These affect the dermis and are classified as superficial (moist, blistered, very painful) or deep (drier, slow blanching, often scarring/contractures) [5].
- Third-degree (TD): These destroy the epidermis and dermis and may injure subcutaneous tissue. The skin may appear white, red, or brown-black. It is dry and hypoesthetic, and healing typically leads to scarring and contractures [5].
- Fourth-degree burns: These extend beyond the skin into deeper tissues such as fascia, muscle, or bone [5].

Timely and appropriate treatment can save lives, shorten hospitalization and recovery time, and reduce the long-term scarring consequences [6]. Burn management requires specialized expertise and immediate access to sufficient resources [7]. Accurate burn classification is essential in the assessment, diagnosis, and treatment planning [8]. In this context, we propose using a Convolutional Neural Network (CNN) [9] for burn image classification to support timely diagnosis and treatment.

Convolutional Neural Networks (CNNs) are deep learning architectures widely used in medical image analysis due to their ability to automatically extract hierarchical spatial features from images [9]. CNN-based approaches have demonstrated strong performance in classification and detection tasks across multiple biomedical imaging applications [9,10,11,12]. Their capability to learn discriminative visual patterns directly from image data makes them particularly suitable for burn severity assessment, where accurate recognition of tissue characteristics is essential.

Table 1 summarizes key studies on burn image severity classification, including the Machine Learning (ML) methods, datasets, and main results.

Suha et al. (2022) [10] analyzed 1530 images comparing traditional machine learning methods with CNNs trained from scratch and via transfer learning; VGG16 achieved the best performance (95.6% accuracy). Ferdinand et al. (2023) [11] evaluated several YOLO (You Only Look Once) architectures and reported a precision of 75.8% using YOLOv5l on a dataset of 3771 images. Yildiz et al. (2024) [12] reported an overall precision of 81% applying YOLOv7 to 7006 images. These studies relied on data augmentation and applied traditional machine learning and deep learning approaches, including pretrained CNNs and YOLO models, across datasets of varying size.

## 2. Materials and Methods

### 2.1. Datasets

A dataset of 467 burn images was collected from a private wound care clinic in Mexico and used as the primary dataset. The images show burns located in different anatomical regions and were acquired during routine clinical care between 2021 and 2022. Initial clinical labels were provided by a wound care specialist before model development. Since the images were collected retrospectively from routine clinical documentation, key acquisition metadata such as the exact post-burn day, camera or device details, imaging distance, lighting conditions, and any standardized photography protocols were not available. No patient identifiers or associated clinical information was provided, and the images did not contain metadata that could enable patient identification. All images were fully anonymized before their use in this study.

Initially, images were labeled as first-degree (2 images), second-degree (324 images), and third-degree (141 images). First-degree burns were excluded because they represented only 0.43% of the dataset and were insufficient for model training.

The remaining images were manually reviewed to exclude blurred or poorly illuminated samples, images in which bandages obscured lesions, and images containing identifiable patient faces, thereby ensuring image quality and patient anonymity. After filtering, 210 second-degree and 30 third-degree images were discarded. The final dataset comprised 225 images: 114 second-degree and 111 third-degree burns. For binary classification, second-degree burns were labeled as class 0 and third-degree burns as class 1.

All images were manually resized to 960 × 540 pixels to focus on the burn region and reduce background noise, such as medical instruments or clinical surroundings.

### 2.2. Dataset Splitting

The dataset was divided into training (80%) and validation (20%) sets. The training set contained 180 images (97 second-degree and 83 third-degree), while the validation set comprised 45 images (17 second-degree and 28 third-degree).

The training set was used for model fitting, while the validation set was used to monitor performance and guide model selection, including hyperparameter optimization, color channel sensitivity analysis, and comparison with transfer learning models.

### 2.3. Independent Test Dataset

To evaluate generalization on unseen data, an independent test dataset was incorporated. Provided by the same clinic, it contains burn images from patients treated in 2025, collected independently from those used for model development.

The test set contains 48 images: 24 second-degree burns (class 0) and 24 third-degree burns (class 1). All images were preprocessed using the same pipeline applied during training and validation.

This dataset was excluded from training, hyperparameter optimization, and channel sensitivity analysis and was reserved exclusively for final performance evaluation, including comparison between the proposed model and transfer learning approaches.

### 2.4. Deep Learning Model

The proposed CNN architecture consisted of convolutional layers followed by Batch Normalization and Rectified Linear Unit (ReLU) activation to improve training stability and gradient propagation. MaxPooling layers with a 2 × 2 window were used to reduce feature map dimensionality and computational cost.

Instead of traditional flattening, a GlobalAveragePooling2D layer aggregated spatial information while reducing the number of trainable parameters before the fully connected classification layer.

To further mitigate overfitting during classification, a Dropout layer was added after each fully connected layer, randomly deactivating neurons during training to promote more robust and generalizable feature representations.

### 2.5. Hyperparameter Search

Hyperparameter tuning was conducted using a sequential stage-wise search strategy due to computational constraints. At each stage, one hyperparameter was varied while the others remained fixed, and the optimal value was retained for subsequent stages. The evaluated search space is summarized in Table 2.

Initially, the number of convolutional layers was evaluated while keeping the remaining hyperparameters fixed (32 filters per convolutional layer, kernel size 3, stride 2, one dense layer with 64 neurons, dropout rate 0.4, and batch size 32), after which the remaining hyperparameters were optimized sequentially within the defined ranges.

Weights & Biases [13] was used to track experiments and monitor performance during hyperparameter optimization.

### 2.6. Model Training

Model training used the Adam optimizer, an adaptive method widely adopted in deep learning for efficiently handling sparse gradients and nonstationary objectives [14]. The binary cross-entropy loss function was employed, as it is standard for binary classification and provides a probabilistic interpretation of model outputs [15].

All models were trained for up to 100 epochs, with validation performance monitored at each epoch. To prevent overfitting and improve convergence, EarlyStopping monitored validation loss with a patience of 10 epochs and restored the weights corresponding to the best validation performance. ReduceLROnPlateau was also applied to adaptively adjust the learning rate based on validation loss, reducing it by a factor of 0.5 after 5 epochs without improvement, with a minimum learning rate of 1 × 10^−7^.

### 2.7. Sensitivity Analysis of Color Channels

A sensitivity analysis was conducted to evaluate the impact of different color channel configurations on the performance of the proposed CNN model. This analysis was motivated by the hypothesis that certain channels may contain more discriminative information for burn severity classification, while reducing input channels could decrease data complexity and improve training efficiency.

Similar approaches have been explored in biomedical imaging studies, where specific spectral representations and the green channel have been reported to provide enhanced tissue contrast and discriminative information for medical image analysis [16,17].

After identifying the optimal CNN architecture through sequential hyperparameter search, the same model was retrained using different color channel inputs extracted from the original images. Evaluated configurations included single channels (red, green, blue), pairwise combinations (red-green, red-blue, green-blue), and full RGB.

### 2.8. Comparative Analysis with Transfer Learning Models

To compare the proposed CNN with pretrained architectures, transfer learning strategies were evaluated using MobileNetV2 [18], VGG16 [19] and ResNet50 [20], all widely used in large-scale image recognition and medical imaging tasks.

For all experiments, input images were represented in RGB, resized to 224 × 224 pixels to match the input size of the evaluated architectures, and normalized using the corresponding preprocess_input functions. ImageNet pretrained weights were used, and the convolutional base was frozen to preserve learned features. The original classification head was replaced with a task-specific architecture consisting of a GlobalAveragePooling2D layer, a Dropout layer with a rate of 0.4, and a single-neuron output layer with Sigmoid activation for binary classification.

All models were trained with binary cross-entropy loss and the Adam optimizer, using batch size 32 and up to 100 epochs. For comparability, the same data split, training configuration, and optimization strategy as the proposed model were applied. EarlyStopping and ReduceLROnPlateau callbacks were incorporated with identical parameter settings for controlled convergence and adaptive learning rate adjustment.

This design ensured that performance differences among models were attributable to architectural characteristics rather than training or evaluation variations.

### 2.9. Model Evaluation Metrics

Model performance was evaluated using standard metrics for binary classification: accuracy, precision, recall, and F1-score [15]. Accuracy measures the proportion of correctly classified samples. Precision represents the proportion of correctly predicted positive cases among all predicted positives, reflecting reliability in identifying third-degree burns. Recall (sensitivity) measures the proportion of actual positive cases correctly identified, indicating the ability to detect third-degree burns. The F1-score, the harmonic mean of precision and recall, provides a balanced measure accounting for both false positives and false negatives.

These metrics were computed consistently across all experiments, including hyperparameter search, color channel sensitivity analysis, and comparisons with transfer learning models. All evaluations used the same data split, with 80% of the dataset for training and 20% for validation, the latter used exclusively for performance monitoring and model selection. Generalization on previously unseen data was assessed only through the independent test dataset, reserved for the final evaluation of the proposed model.

## 3. Results

### 3.1. Results of Hyperparameter Optimization and Final Model Selection

**Figure 1:**
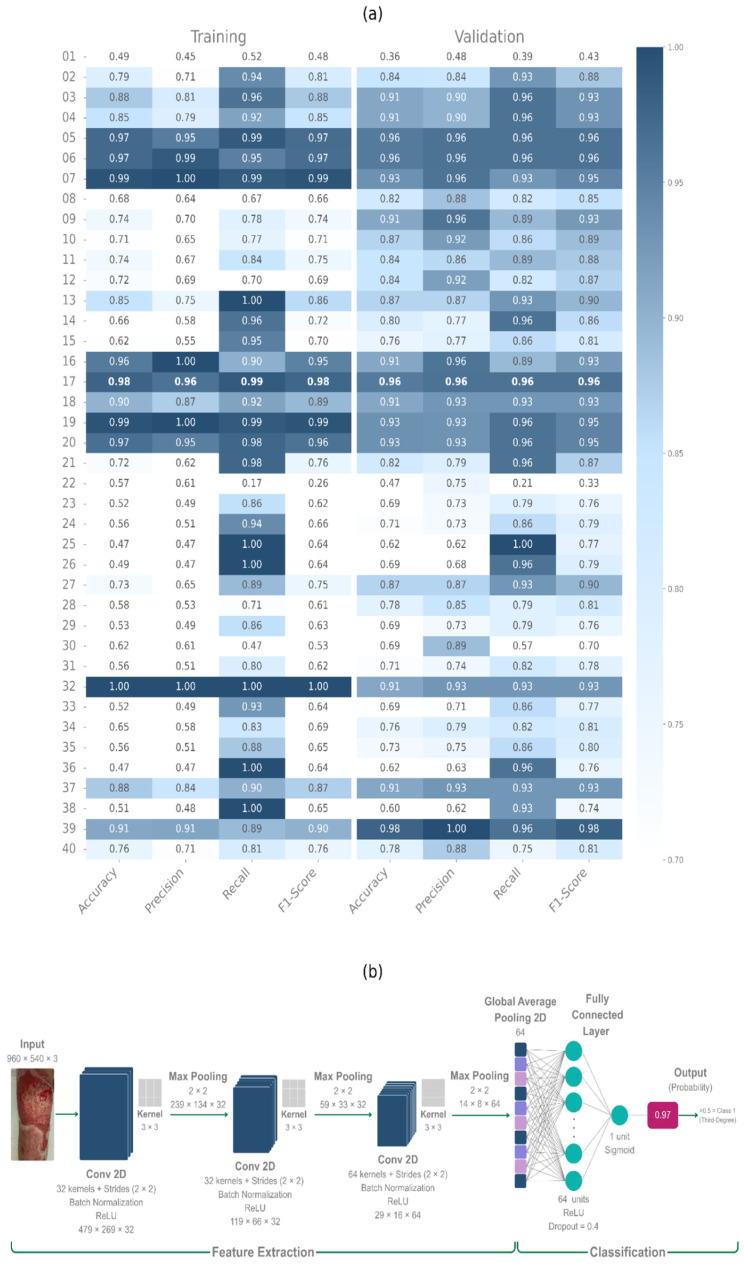
Hyperparameter optimization and final model selection. (**a**) Performance metrics on the training and validation sets for each model were evaluated during the hyperparameter search. (**b**) Architecture of the convolutional neural network corresponding to the best model found.

Sequential hyperparameter optimization yielded a compact CNN with stable validation performance. The selected configuration includes three convolutional layers (32, 32, and 64 filters) with a kernel size of 3 and a stride of 2, providing the most robust results. The evaluated configurations correspond to the combinations of hyperparameters described in Table 2 and indexed from 1 to 40 in Figure 1a.

The classification stage employed a single dense layer with 64 neurons. Regularization experiments identified a dropout rate of 0.4 as optimal for balancing performance and generalization, while a batch size of 32 was most effective for training.

Figure 1a shows a heatmap summarizing performance across all evaluated CNN configurations. Performance generally improved with greater depth and representational capacity, particularly in three-layer convolutional architectures with balanced filters, whereas shallower models or those with limited feature extraction achieved lower accuracy and F1-score, especially on validation data.

Some configurations achieved high training performance but deteriorated on validation, indicating overfitting. Notably, a stride-3 variant of the selected model achieved higher training metrics but poorer validation performance, whereas stride 2 consistently ensured a better training-validation balance and improved generalization.

Overall, architectures combining three convolutional layers, kernel size 3, and stride 2 delivered the most stable performance, underscoring the importance of balancing capacity and generalization rather than maximizing training accuracy alone.

Using these hyperparameters, the final CNN architecture, illustrated in Figure 1b, comprises three convolutional blocks with Batch Normalization, ReLU activation, and MaxPooling, followed by GlobalAveragePooling2D and a 64-neuron dense layer. The output layer consists of a single Sigmoid neuron that predicts the probability of a third-degree burn classification.

### 3.2. Results of Color Channel Sensitivity Analysis

The optimal architecture identified through hyperparameter optimization was retrained on the seven color channel configurations under the same training conditions used previously.

**Figure 2:**
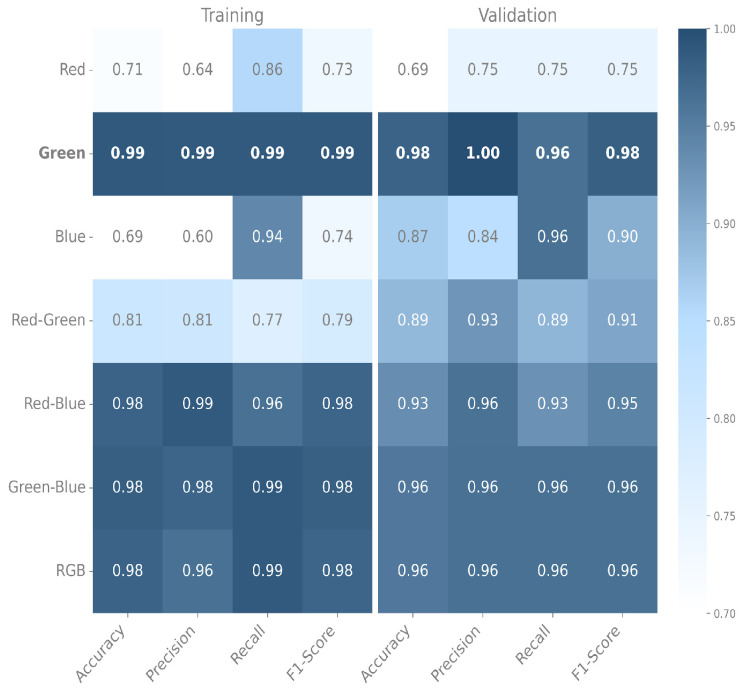
Model performance metrics on the training and validation sets for each color channel combination.

Figure 2 summarizes training and validation performance across channel configurations. The model trained using only the green channel achieved the best overall results, obtaining the highest validation accuracy (0.98), precision (1.00), recall (0.96), and F1-score (0.98). Compared with the next-best configurations (green-blue and RGB), it improved the F1-score by approximately 2 percentage points and outperformed the weakest configuration (red channel) by more than 23 percentage points.

Although the green-blue configuration showed a slightly higher accuracy, precision, and F1-score than RGB on training data (with identical recall), this advantage did not translate into validation improvements. The single green-channel model therefore achieved a better training-validation balance, indicating superior generalization.

Additionally, using a single-color channel reduces input dimensionality and computational complexity, making the green-channel configuration a more efficient and parsimonious solution for burn image classification.

Previous reports have highlighted the discriminative capability of the green channel in biomedical imaging [17].

### 3.3. Results of Performance Evaluation of the Proposed Model

**Figure 3:**
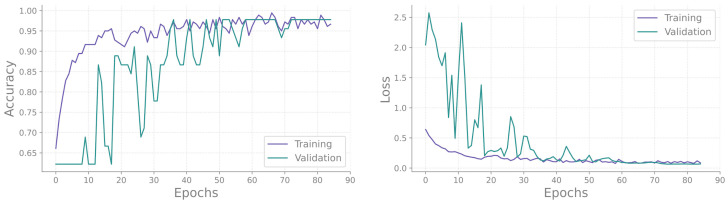
Learning curves showing accuracy and loss on the training and validation sets for the proposed model.

Following the analysis of color channel sensitivity, the model trained using only the green channel was selected for final evaluation. Figure 3 shows the training and validation learning curves. During approximately the first 30 epochs, validation accuracy and loss fluctuated, stabilizing around epoch 35 and closely tracking the consistently stable training curves. In later epochs, the close alignment of the curves indicates the absence of overfitting and stable generalization on validation data. Training was set for 100 epochs but stopped at epoch 85 via EarlyStopping, while ReduceLROnPlateau reduced the learning rate to 1.5625 × 10^−5^.

**Figure 4:**
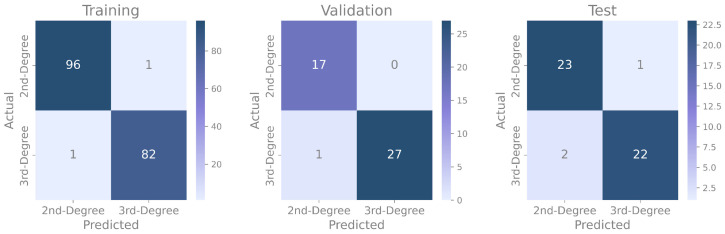
Confusion matrices of the proposed model for training, validation, and test sets.

Figure 4 presents confusion matrices for training, validation, and independent test datasets. On the training set, only two images were misclassified, one from each class, indicating strong discrimination between both degrees of burns. Class-wise analysis shows nearly identical performance across the two classes, with accuracy, precision, recall, and F1-score values close to 0.99, suggesting no class-specific bias.

On the validation set, performance remained comparable, with a single misclassification where one third-degree burn was predicted as second-degree. Class-wise metrics remained balanced, with second-degree burns obtaining values between 0.94 and 1.00 and third-degree burns between 0.96 and 1.00 across all metrics.

On the independent test dataset, performance decreased slightly relative to training and validation but remained high. The confusion matrix shows three errors: one second-degree burn predicted as third-degree and two third-degree burns predicted as second-degree. Nevertheless, class-wise metrics remained balanced, with accuracy, precision, recall, and F1-score ranging from 0.92 to 0.96 across both classes, confirming robust generalization without class bias.

Overall, the proposed model maintains stable and unbiased classification performance across training, validation, and independent test datasets.

### 3.4. Results of Comparative Analysis with Transfer Learning Models

**Figure 5:**
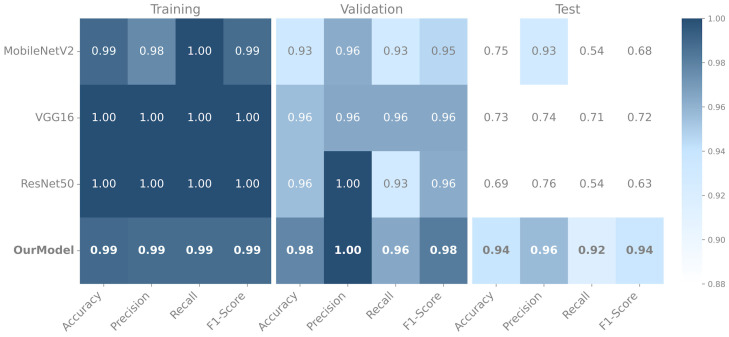
Comparison of performance metrics between the proposed model and transfer learning approaches (MobileNetV2, VGG16, and ResNet50).

Figure 5 compares the performance of the transfer-learning models with the proposed CNN on the training, validation, and independent test datasets.

All pretrained architectures achieved training metrics between 0.98 and 1.00. On the validation set, MobileNetV2 showed reduced performance, with an accuracy and recall of 0.93. VGG16 remained consistent, reaching 0.96 across all validation metrics. ResNet50 exhibited a decrease in recall (0.93) while preserving values between 0.96 and 1.00 for the remaining metrics.

Performance differences became more evident on the independent test set, suggesting limited generalization. MobileNetV2 achieved 0.75 accuracy; although precision remained high (0.93), recall dropped to 0.54, indicating that nearly half of the third-degree burn images were misclassified as second-degree. VGG16 reached 0.73 accuracy, while ResNet50 recorded the lowest accuracy (0.69). Overall, F1-scores ranged from 0.63 to 0.72.

In contrast, the proposed model maintained strong performance on the independent test set (accuracy 0.94, precision 0.96, recall 0.92, and F1-score 0.94), demonstrating superior generalization compared with transfer learning approaches, while using a compact and computationally efficient architecture for second- and third-degree burn image classification.

### 3.5. Implementation and Graphical User Interface

The proposed CNN model and transfer learning models (MobileNetV2, VGG16, and ResNet50) were integrated into a graphical user interface developed with the Gradio library, enabling users to upload a burn image for automatic analysis.

This application allows users to select a model and automatically preprocess the input image according to the selected architecture. For the proposed model, the image is resized to 960 × 540 pixels, and only the green color channel is extracted, resulting in a single-channel input representation. For the transfer learning models, the image is represented in RGB format, resized to 224 × 224 pixels, and processed using the corresponding preprocessing function associated with each architecture. The interface displays the predicted class (second-degree or third-degree burn) along with the associated prediction probability.

This interface enables rapid evaluation of burn images using multiple deep learning approaches within a single application and is publicly available (see [21]).

An interactive implementation of the proposed model is publicly available through a Hugging Face Space interface. https://huggingface.co/spaces/tit-for-tat/Second-and-Third-Degree-Burn-Image-Classifier (accessed on 20 May 2026).

## 4. Discussion

The results of this study show that the proposed model is an effective alternative for classifying second- and third-degree burn images. Compared with previous works, our approach achieves strong performance while maintaining a compact architecture, balancing accuracy and computational cost.

YOLO-based approaches [11,12] have reported lower performance (75.8–81% precision) than those obtained in the proposed approach; these schemes incorporate additional detection and segmentation components that increase system complexity and computational requirements. However, Suha et al. (2022) [10] achieved high performance (95.6% accuracy) using transfer learning, which typically relies on deeper pretrained models and greater computational cost.

Interestingly, despite their substantially larger representational capacity, the transfer learning architectures evaluated in this study showed a more pronounced performance decrease on the independent test dataset, suggesting reduced generalization capability compared with the proposed compact CNN.

Table 3 presents a comparative analysis of previous burn image classification and detection studies and the proposed approach, including evaluation strategies, generalization assessment, and key methodological features.

In contrast to previous studies, the proposed model was evaluated using training, validation, and independent test datasets. Although the model achieves 94% accuracy on the independent test dataset, the consistency across datasets indicates the absence of overfitting and confirms its generalization capability to unseen data. This is particularly relevant in medical imaging studies with limited datasets, where overfitting remains a common challenge in deep learning applications.

Additionally, the proposed model achieves competitive performance using a lightweight architecture trained from scratch and relying exclusively on the green channel. This combination reduces input dimensionality and computational complexity while maintaining strong generalization performance. These findings suggest that increasing architectural complexity does not necessarily translate into improved performance for limited clinical image datasets.

Unlike most related studies, the proposed model was integrated into an interactive graphical user interface publicly available on Hugging Face, facilitating practical evaluation in clinical and educational environments. Supporting materials, including trained models and notebooks, are also available through a Zenodo repository [21].

This study has limitations. First, the retrospective collection of dataset images during routine clinical care means that information about the camera or device used, lighting conditions, imaging distance, and standardized acquisition protocols were not available. Second, the approach is restricted to binary classification between second- and third-degree burns. Future work should validate the approach using larger and more diverse datasets and extend the framework toward multiclass classification, including all four burn degrees.

## 5. Conclusions

This study presents a CNN for automatic classification of second- and third-degree burn images using clinical data. Through hyperparameter optimization and color channel sensitivity analysis, a green-channel-only model achieved strong and balanced performance on training, validation, and independent test datasets.

The results show that competitive performance can be obtained without complex transfer learning models. A lightweight architecture trained from scratch achieved high accuracy while reducing input complexity and computational cost without compromising generalization.

Data quality is critical for training artificial intelligence models. In this study, image preprocessing was essential to achieving the reported results. By removing elements that introduced noise (such as clinical backgrounds or medical instruments) and focusing on the burn area along with a small region of healthy skin, the model was able to generalize unseen data by learning representative burn patterns rather than contextual artifacts.

The final model was integrated into a graphical user interface, enabling practical use for burn image classification and demonstrating its potential as a support tool in clinical and educational environments.

Future work should include larger and more diverse datasets and extend the approach toward multiclass classification covering additional burn degrees. Overall, these findings indicate that compact, well-optimized deep learning models can provide efficient and reliable solutions for burn severity classification.

## Figures and Tables

**Table 1:** Summary of related studies addressing burn image classification and detection.

Reference	Methods	Original Database Size	Main Findings
[10]	Classical ML models and CNNs with transfer learning (VGG16, MobileNet, and ResNet50).	1530 images.	Best performance achieved by VGG16 (95.6% accuracy).
[11]	YOLOv5m, YOLOv5l, YOLOv5x, and YOLOv7.	3771 images.	Best performance achieved by YOLOv5l (75.8% precision).
[12]	YOLOv7	7006 images.	Best performance achieved by YOLOv7 (81% precision).

**Table 2:** The hyperparameter search space is evaluated during the sequential stage-wise tuning process.

Hyperparameter	Search Space
Number of convolutional layers	1, 2, 3
Number of filters per convolutional layer	16, 32, 64
Kernel size	3, 5
Stride	1, 2, 3
Number of dense layers	1, 2, 3
Number of neurons per dense layer	32, 64, 128
Dropout rate	0.3, 0.4, 0.5
Batch size	16, 32, 64

**Table 3:** Comparative analysis of burn image classification and detection studies.

Reference	Best-Performing Model	Generalization Assessment	Key Findings and Features
[10]	VGG16 transfer learning.	Test performance reported (95.6% accuracy).	High classification performance achieved using transfer learning and RGB images.
[11]	YOLOv5l	Validation: 76.6% precision; test: 75.8% precision.	Object detection approach with hyperparameter tuning and RGB images.
[12]	YOLOv7	Overall test performance reported (81% precision).	Integrated mobile application for burn image detection using RGB images.
This work	Compact CNN trained from scratch.	Training: 99% accuracy; validation: 98% accuracy; independent test: 94% accuracy.	Compact single green-channel model with strong generalization, reduced computational complexity, and an easy-to-use GUI publicly available on Hugging Face.

## Data Availability

Supporting materials, including trained models, a Gradio inference interface, and Jupyter notebooks for experiments and evaluation, are available in a Zenodo repository at https://doi.org/10.5281/zenodo.18870161 [21].

## Abbreviations

The following abbreviations are used in this manuscript:

TBSA	Total Body Surface Area
FD	First-degree
SD	Second-degree
TD	Third-degree
CNN	Convolutional Neural Network
YOLO	You Only Look Once
ReLU	Rectified Linear Unit
RGB	Red, Green, Blue
Conv2D	Two-dimensional convolutional layer

## References

[1] World Health Organization Burns. https://www.who.int/news-room/fact-sheets/detail/burns.

[2] Secretaría de Salud, Secretariado Técnico del Consejo Nacional para la Prevención de Accidentes (2016). Modelo para la Prevención de Quemaduras en Grupos Vulnerables en México.

[3] Rossella E., Giulio M., Michele M. (2022). Burns: Classification and treatment. Textbook of Plastic and Reconstructive Surgery: Basic Principles and New Perspectives.

[4] Warby R., Maani C.V. (2023). Burn classification. StatPearls.

[5] Vidaurri de la Cruz H., González Gaytán D., Villela Segura U., Castellanos Castro J.L., Valencia Herrera A.M., Pineda Lemus M.C., Arvizu Ramírez F., Godínez Chaparro J.A., Bueno Wong J.L., Bolaños Aguilar M.A. (2022). SAM. Heridas y Cicatrización.

[6] Żwierełło W., Piorun K., Skórka-Majewicz M., Maruszewska A., Antoniewski J., Gutowska I. (2023). Burns: Classification, Pathophysiology, and Treatment: A Review. Int. J. Mol. Sci..

[7] Hughes A., Almeland S.K., Leclerc T., Ogura T., Hayashi M., Mills J.A., Norton I., Potokar T. (2021). Recommendations for burns care in mass casualty incidents: WHO Emergency Medical Teams Technical Working Group on Burns (WHO TYOLOWGB) 2017–2020. Burns.

[8] Moreno Arjol I., Vargas Escuer M.E., Fernández Álvarez A., Embid Sáez G., Cantín Barrera R., Castro Pueyo J. (2021). Revisión bibliográfica sobre quemaduras en atención primaria: Clasificación y abordaje. Rev. Sanit. Investig..

[9] Zhao X., Wang L., Zhang Y., Han X., Deveci M., Parmar M. (2024). A review of convolutional neural networks in computer vision. Artif. Intell. Rev..

[10] Suha S.A., Sanam T.F. (2022). A deep convolutional neural network-based approach for detecting burn severity from skin burn images. Mach. Learn. Appl..

[11] Ferdinand J., Viriya Chow D., Yuda Prasetyo S. (2023). Automated skin burn detection and severity classification using YOLO Convolutional Neural Network Pretrained Model. E3S Web Conf..

[12] Yıldız M., Sarpdağı Y., Okuyar M., Yildiz M., Çiftci N., Elkoca A., Yildirim M.S., Aydin M.A., Parlak M., Bingöl B. (2024). Segmentation and classification of skin burn images with artificial intelligence: Development of a mobile application. Burns.

[13] Biewald L. Experiment Tracking with Weights and Biases. https://wandb.ai.

[14] Kingma D.P., Ba J. Adam: A Method for Stochastic Optimization. Proceedings of the International Conference on Learning Representations (ICLR).

[15] Goodfellow I., Bengio Y., Courville A. (2016). Deep Learning.

[16] Çelik Y. (2026). Deep Learning-Based Detection of Bowel Sound Events in Continuous Recordings. Sci. Rep..

[17] Khurshid M., Chiranjeev C., Singh R., Vatsa M. (2026). Classifying Retinal Images via Vascular-Optic Disc Cross-Segmentation and Attentive Feature Selection. Sci. Rep..

[18] Sandler M., Howard A., Zhu M., Zhmoginov A., Chen L.C. MobileNetV2: Inverted Residuals and Linear Bottlenecks. Proceedings of the IEEE Conference on Computer Vision and Pattern Recognition (CVPR).

[19] Simonyan K., Zisserman A. (2015). Very Deep Convolutional Networks for Large-Scale Image Recognition. arXiv.

[20] He K., Zhang X., Ren S., Sun J. Deep Residual Learning for Image Recognition. Proceedings of the IEEE Conference on Computer Vision and Pattern Recognition (CVPR).

[21] Montecinos-Rodríguez Y., Torres-Santana F.J., Díaz-González L. (2026). Automated Classification of Second- and Third-Degree Burn Images Using Convolutional Neural Networks. Zenodo. https://zenodo.org/records/18870162.

